# Lifestyle factors in preventing mental health disorders: an interview with Felice Jacka

**DOI:** 10.1186/s12916-015-0490-5

**Published:** 2015-10-14

**Authors:** Felice N. Jacka

**Affiliations:** IMPACT Strategic Research Centre, School of Medicine, Deakin University, Geelong, 3220 Victoria Australia

**Keywords:** Anxiety, Depression, Diet, Physical activity, Prevention, Psychiatry

## Abstract

In this video Q&A, we talk to Associate Professor Felice Jacka about population health approaches to the primary prevention of mental disorders across the lifespan. These include addressing lifestyle factors, such as diet, smoking and physical activity. Latest strategies are being developed through epidemiological studies and clinical trial evidence. Challenges in preventing mental disorders in general and specifically in the workplace are discussed, together with future directions on promoting well-being.

**Electronic supplementary material:**

The online version of this article (doi:10.1186/s12916-015-0490-5) contains supplementary material, which is available to authorized users.

## Introduction

Associate Professor Felice Jacka is a Principal Research Fellow at Deakin University, with honorary positions at the Murdoch Children’s Research Institute, The University of Melbourne and the Black Dog Institute. She is President of both the International Society for Nutritional Psychiatry Research (ISNPR) and the Australian Alliance for the Prevention of Mental Disorders (APMD). Her ongoing program of research focuses on lifestyle behaviours, particularly diet, as risk factors for the common mental disorders, depression and anxiety. She is internationally recognised as a pioneering researcher in the new discipline of nutritional psychiatry, leading multiple studies establishing associations between diet quality and mental health in adults, adolescents and children from many countries. She has also developed a theoretical framework for this research, which has extensive applications and implications for public health and clinical practice. Associate Professor Jacka is the recipient of a number of grants and awards, including funding for the first randomised controlled trial (RCT) of dietary improvement as a treatment strategy in major depression. Her research program comprises a broad range of observational investigations, as well as the development and evaluation of community-based and clinical interventions. Her goals are to develop effective, best practice strategies for the universal primary prevention of the common mental disorders, as well as effective secondary prevention and treatment strategies for those affected.

In this interview (Video Q&A: Additional file 1), we talk to Associate Professor Felice Jacka about how lifestyle factors, such as diet, smoking and physical inactivity, can contribute to common mental health disorders, like depression and anxiety, across different population groups. We discuss the latest strategies and investigations that are addressing the issue and the future prospects associated with prevention of mental disorders.

## Edited transcript

### Tell us a bit about yourself and how you became involved in population health and mental health

I guess it was through quite an unconventional route. My first degree was in fine art and I was an artist. But I had always had a very strong interest in mental health and also a personal awareness of nutrition. When I came into psychiatry research, I was intrigued to see that there really was not an evidence base around the links between nutrition and mental health. That surprised me, I guess, because we know that nutrition is very important for many diseases that are comorbid with depression, such as cardiovascular disease and obesity. That led me down this path of investigation.

Many of the research findings that we have uncovered lend themselves to prevention and public health. Also, as a pragmatist, it always seems intriguing to me that so much money is spent on treatment, and almost nothing spent on prevention. That seems like a very inefficient use of resources.

### Can you describe the population health approach to the primary prevention of common mental disorders (depression and anxiety)?

Previously in prevention research, there have been a number of studies and programs with a very good evidence base that are focussed on what we call “selected and indicated prevention”. Selected is when you target people who are at elevated risk for mental disorders and the term “indicated prevention” refers to when you target people who already have elevated symptoms. With a universal approach to prevention, you are really targeting everybody. The advantages for that are that you don’t then need to screen people, which can be very expensive. You are also not creating barriers for people who may not want to seek help because of stigma.

We know now that there are many good ways of preventing mental disorders at the population level, right across the life course. We know, for example, that if you have behavioural interventions, social and emotional learning, or programs that target parenting and support parents, you may be able to mitigate some of the child abuse and neglect that we know is a really strong risk factor for mental disorders across the lifespan. Similarly, in schools one could target bullying, which we also know is an important risk factor. There are many studies that suggest that cognitive behavioural therapy programs are useful for school children, and of course they can be delivered online now, which is another useful way of targeting and delivering these interventions. There is a good evidence base for workplace mental health programs that aim to increase resilience and reduce stress. Then, of course, in older age groups, increasing social connectedness and social support can be useful in preventing mental health problems. So there are many interventions that we know have a good evidence base that can be applied at the population level, and that is what we are focussing on promoting now.

New evidence around the importance of diet and nutrition to the genesis and the progression of common mental disorders, accompanied by what we already know about physical activity and how important that is in both preventing and treating mental disorders, such as depression, is also emerging. Thus we can really start to think about population-based approaches that are integrated with the strategies that have already been implemented for obesity, cardiovascular disease, and a whole range of other noncommunicable diseases (NCDs) that we know are co-morbid with depression. In this way we can increase efficiency and leverage on what is already being done in that field.

One of the key recommendations is thus to start measuring mental health outcomes in such population-level health interventions. Thus we are not just focussed on metabolic outcomes and associated NCDs but we are also measuring mental health.

### Discuss how an unhealthy diet can worsen mental health outcomes

What we have seen now, 4 or 5 years after the first studies on this topic were published, is that there is definitely a relationship between diet quality and common mental disorders, and it seems to exist right across the life course. We started off investigating this in adults, but since then we have done a lot of work looking at this question in adolescents, in older people, and more recently really looking at the influence of early life nutritional exposures to mental health outcomes in children.

The evidence seems pretty clear. There is a relationship between nutrition and mental health across the life course. Of course, understanding how those relationships work is our next task and this is something that we are starting to focus on now. For example, a lot of the data at this point are coming from experimental models, which are investigations in animals that look at the direct impact of high-fat refined sugar foods, or “junk foods”, on a whole range of parameters in the brain that we know are relevant to mental health. The effects are not just in the brain, but more systemically, such as inflammation and oxidative stress.

We know that these sorts of foods are very toxic to the brain as they reduce proteins, such as brain-derived neurotrophic factor (BDNF), and they impact on synaptic plasticity, learning and memory, and also up-regulate the stress response, immune and oxidative stress systems. We are also very interested in the role of the microbiota, particularly the gut microbiota, and how this mediates environmental exposures, such as diet, and their relationships to mental health and other health outcomes. We think that the gut is a really key part of that pathway.

### What are the latest strategies that are being developed to promote the prevention of mental disorders?

In Australia, there have been two initiatives – one that is Australian-focussed and one that is more international. In 2013 in Australia, we initiated the Alliance for the Prevention of Mental Disorders (APMD). This is aiming to progress the agenda and the discussion in Australia about the need to take prevention more seriously so that more funding and research energy contributes towards it. Repeated stakeholder surveys have suggested that prevention is of the highest priority for many people. However, less than 3 % of the research funding goes to prevention, and fewer than 3 % of papers in the field are on prevention.

Given the enormous burden of illness associated particularly with depression and common mental disorders, a group of us felt that we really needed to push this forward in 2013. It comprises many of the most senior researchers in Australia who are interested in prevention and actively working in this area, plus representatives from many of the non-government organisations and representative councils. Together, we really want to get this conversation moving, advocate for more research funding for the field, increase capacity in the area, and get more people talking about this.

More widely, in 2013 we launched the International Society for Nutritional Psychiatry Research (ISNPR). This currently has about 200 members from right across the globe and is becoming more and more active. This society is aiming to increase the amount of research that is being done in the field, and raise capacity and collaborations to have this topic better represented in psychiatry research and practice. This lends itself to the population health approach to both prevention and treatment.

### How does the Alliance for the Prevention of Mental Disorders (APMD) compare with other prevention initiatives?

There are a number of initiatives in different countries. Some of them are part of government departments and they are very well funded. One particular one in the US really focuses on substance abuse; others in the European jurisdiction focus on workplace mental health. There is the European Network for Mental Health Promotion (ENMHP), which probably is more closely aligned with what we are trying to do in Australia.

Our alliance is very new, so one of the key things we need to focus on in the next 12 months is to look at funding strategies so that we can increase the capacity for us to act. This may include lobbying, advocacy and administrative support. We would like to become a focal point whereby we can provide resources to policymakers and local councils who are wanting to do some prevention and promotion work. We want to become the port of call to support and promote these sorts of programs.

### The “SMILES” trial on the dietary intervention for adults with major depression is underway. Can you comment on when the results are expected and how this will influence future research?

Since the end of 2009–2010, there has been an exponential increase in the number of studies that have looked at the links between diet and mental health, but they are largely observational in nature. We have had two really interesting studies in the last 12 months that have suggested the prevention of depression by improving diet. However, to date there have been no studies that show whether if you are already depressed and you improve your diet, this results in an improvement in mood.

We are running the first study to our knowledge that really tests this empirically. It is a clinical trial where people are randomised to receive either a quite detailed dietary counselling and support, or a social support condition. We hope to have the results by the end of 2015 and the study is underway as we speak.

Importantly with this, we are collecting a host of biological samples from the participants. We want to know what happens in the body if you change diet and whether these changes relate, then, to changes in mood. Stool, blood and saliva samples are being collected, so we can look at not only things such as nutrient levels, but also inflammatory and oxidative stress markers, the microbiota, very importantly, and also cortisol levels. These may change when there is a change in diet and then, hopefully, relate to improvements in mental health.

### Describe how diet quality affects the lifespan from early life to old age

We looked at the very early life question recently in a very large sample of Norwegians. We had data for more than 23,000 mothers and their children, participating in the ongoing Norwegian Mother and Child Cohort Study (MoBa). We looked at what mums ate when they were pregnant and also what children ate in the first few years of life, and then the children’s mental health outcomes. Thus internalising and externalising behaviours over the years to 5 years of age were determined. We saw very clear relationships between both mothers’ diets and the children’s diets and the internalising and externalising behaviours.

There has been more work done in this field since then. The Generation R cohort study showed quite a similar set of findings. Then of course we have also done quite a bit of work in adolescents and showed both cross-sectionally and prospectively that diet is associated with depression in adolescence. Given that common mental disorders have a very early age of onset, we think that this is highly relevant. We were able to take into account things such as family functioning and poor family management as these may influence the relationship. In the last 4 or 5 years, we have seen these associations right across countries, across cultures and across age groups. Thus we are very comfortable in saying that that relationship does exist. We now need to understand the mechanism of this association so we can target our interventions.

### What are the current research approaches to address other lifestyle factors, such as smoking and physical activity, on mental disorders?

It is very interesting to consider, over the past 2 years, the research around smoking cessation. This is because people with mental disorders are far more likely to smoke than people without mental disorders. I think in clinical care there has been this assumption that encouraging people to give up smoking will actually add to their stress levels or maybe worsen their mental health symptoms as they withdraw from tobacco. However, a large systematic review has recently showed that people who give up smoking experience an improvement in mental health and well-being. Smoking, in people with mental disorders, is associated with an increased likelihood of suicide, of worse treatment outcomes and a poorer prognosis. Thus we think that it is very important that smoking cessation is targeted as a key clinical aspect of care.

Similarly, physical activity, we have known for quite a long time now, is important, particularly in the risk for depression. People who are physically inactive are at increased risk of depression, whereas people who are physically active are protected and have a reduced risk. Exercise is also a very useful treatment strategy in depression. However, to my knowledge – certainly in Australia – those key understandings have not yet made their way into official clinical guidelines for treatment of mental disorders. I think it is very important, not just from a primary prevention point of view, but from secondary and tertiary prevention, that diet, physical activity and smoking are all targeted as key aspects of health for people with mental disorders, as well as those who are at risk for physical disorders.

### Describe the challenges in preventing mental disorders in general and in the workplace

Prevention is a really difficult topic for many reasons. One, of course, relates to policymakers and politicians, and the second relates to funding bodies in research. The costs for prevention are incurred up-front but the payoff is quite a long way down the track. This means it is less appealing in terms of getting re-elected, etc. This is a particular challenge but, of course, even though governments change in the short term, government bodies, councils and people are still going to be around in 10 or 15 years’ time. So whilst it is important to be able to show short-term benefits, there is still a real importance to be placed on long-term outcomes and there needs to be a policy framework for long-term outcomes in prevention.

Similarly, in the workplace, time and costs are issues for businesses. However, mental disorders account for quite a large percentage of the burden of sickness absence, lost productivity, etc. We know that many of these interventions in the workplace, aiming to improve depression, are both effective and cost-effective. So there is an enormous amount to be gained for businesses by focussing on this and implementing some of these evidence-based prevention programs in the workplace.

### What do you think are the future directions of preventing mental disorders and promoting well-being?

In Australia, we think it is time for a national strategy for the prevention of mental disorders. This is something we are strongly advocating. It is also critical that policymakers start to fund things that have an evidence base, and not continually fund research that does not have an evidence base. I think that is a common issue across countries.

We are hoping to soon be able to model the short-, medium- and long-term cost effectiveness of preventative interventions, similar to what the Department of Health presented in the Knapp report, *Mental health promotion and prevention: The economic case* [1]. This is one of the things that I think may make a difference. If you can show how much money, potentially, can be saved in the long term, as well as the short and medium term, I think that that strengthens the argument for prevention. It is also really important to focus on the cost of not doing it. Thus to show that not addressing these things results in short-, medium- and long-term costs. That is really quite a potent message as well. We also need to communicate this to the public because if they understand that it is possible to prevent mental disorders, they are more likely then to support government initiatives, even if the payoff is in the long term.

I think it is important that we take a life course approach to resilience. One of the challenges is that so many of the environmental factors that impinge on the risks for mental disorders happen outside of the mental health sector. Thus it really does need a whole population approach. We need to protect people from vulnerability risk factors, such as child abuse and neglect, poverty, bullying, workplace stress, and social isolation, and target these by building resilience through education, social and emotional learning, and community-based interventions. Volunteering is a really effective way of building social connectedness. Improving physical health and health behaviours, such as diet, physical activity and smoking, is really important.

There are many prevention options that we know have a good evidence base and that can be implemented right across the life course. These include parenting, early life, in the school system, outside in the community and in older age. We really need to be thinking locally and acting at the community level, but having a framework that is set at the national level.

None of these things are impossible, but we do need political will and we need the community to get behind that. We would hope that in the future, this life course approach, this whole strategy for prevention and mental health promotion – at every stage of life and in every setting – is developed and taken seriously.

### Where can I find out more?

See references [1–10].

## Additional file

Additional file 1:
**Audiovisual file - interview with A/Prof Felice N Jacka.** (MP4 56914 kb)

## Abbreviations

APMD: Alliance for the Prevention of Mental Disorders
BDNF: Brain-derived neurotrophic factor
ENMHP: European Network for Mental Health Promotion
ISNPR: International Society for Nutritional Psychiatry Research
MoBa: Norwegian Mother and Child Cohort Study
NCD: Noncommunicable disease
RCT: Randomised controlled trial
